# Involvement of trehalose in hydrogen sulfide donor sodium hydrosulfide-induced the acquisition of heat tolerance in maize (*Zea mays* L.) seedlings

**DOI:** 10.1186/1999-3110-55-20

**Published:** 2014-02-03

**Authors:** Zhong-Guang Li, Li-Ju Luo, Li-Ping Zhu

**Affiliations:** 1 School of Life Sciences, Yunnan Normal University, Kunming, 650092 PR China; 2 Engineering Research Center of Sustainable Development and Utilization of Biomass Energy, Ministry of Education, Kunming, 650092 PR China; 3 Key Laboratory of Biomass Energy and Environmental Biotechnology, Yunnan Province, Yunnan Normal University, Kunming, 650092 PR China

**Keywords:** Heat stress, Heat tolerance, Hydrogen sulfide, Maize (*Zea mays* L.) seedlings, Trehalose

## Abstract

**Background:**

Trehalose, a non-reducing disaccharide, which involves in the acquisition of various stress tolerance, while hydrogen sulfide (H_2_S) is considered as a cell signal molecule, but H_2_S-induced heat tolerance and involvement of trehalose in plants is still unclear.

**Results:**

In present study, pretreatment with hydrogen sulfide donor sodium hydrosulfide (NaHS) markedly increased the accumulation of endogenous H_2_S in maize seedlings under normal culture conditions, which in turn improved survival percentage of maize seedlings and mitigated increase in electrolyte leakage and malonaldehyde (MDA) accumulation under heat stress. In addition, treatment with NaHS activated increase in the activity of trehalose-6-phosphate phosphatase (TPP) under normal culture conditions, followed by induced the accumulation of endogenous trehalose, but this accumulation was eliminated by addition of sodium citrate, an inhibitor of TPP. During the process of heat stress, maize seedlings treated with NaHS maintained higher TPP activity and trehalose content than those of control. On the other hand, exogenous application of trehalose also increased the content of endogenous trehalose in maize seedlings under normal culture conditions, alleviated increase in electrolyte leakage and MDA accumulation under heat stress, which in turn improved survival percentage of maize seedlings, and the heat tolerance induced by trehalose was enhanced by exogenous supplement of NaHS, but exogenous trehalose treatment had not significant effect on the accumulation of endogenous hydrogen sulfide in maize seedlings.

**Conclusion:**

These data suggest that sodium hydrosulfide pretreatment could improve heat tolerance of maize seedlings and this improvement may be involved in trehalose accumulation by activating TPP activity.

**Electronic supplementary material:**

The online version of this article (doi:10.1186/1999-3110-55-20) contains supplementary material, which is available to authorized users.

## Background

Trehalose, a stable non-reducing disaccharide, which is composed of two D-glucopyranose units with an α,α (1 → 1) linkage, it can withstand heating at 100°C between pH 3.5–10 for 24 h. The α,α-1,1 configuration is crucial for the ability of trehalose to preserve lipid bilayer structure in the absence of water (Goddijn and van Dun, 1999; Iordachescu and Imai, 2008; Paul et al., 2008; Fernandez et al., 2010). Trehalose has been shown to efficiently stabilize dehydrated enzymes, proteins and lipid membranes, as well as protect biological structures from damage during desiccation by replacing water. Trehalose forms an amorphous glass structure that limits molecular motion, preventing protein aggregation and scavenging free radical under stress conditions (Goddijn and van Dun, 1999; Benaroudj et al., 2001; Iordachescu and Imai, 2008; Paul et al., 2008; Fernandez et al., 2010). Based on these unique features and functions, trehalose metabolism and signaling turns into an area of emerging significance. Trehalose is found in various organisms, including bacteria, fungi, yeast, algae, insects and some plants (Paul et al., 2008; Fernandez et al., 2010). To date, two pathways of trehalose metabolism were identified: the trehalose-6-phosphate synthase/phosphatase (OtsA-OtsB) pathway and the trehalase pathway. The former is used for trehalose synthesis, whereas the latter is proposed to operate in trehalose degradation. In plants, the OtsA-OtsB pathway is the only biosynthesis pathway found. Trehalose 6-phosphate synthase (TPS) catalyzes the transfer of glucose from uridine diphosphate glucose (UDPG) to glucose 6-phosphate (G6P) to form trehalose 6-phosphate (T6P) and uridine diphosphate (UDP), and trehalose phosphate phosphatase (TPP) dephosphorylates T6P to form trehalose and inorganic phosphate. Trehalase is the key enzyme responsible for the hydrolysis of trehalose (Goddijn and van Dun, 1999; Iordachescu and Imai, 2008; Paul et al., 2008; Fernandez et al., 2010; Zhou et al., 2013). Therefore, the control of TPP and trehalase is key in the accumulation of endogenous trehalose in plants (Paul et al., 2008). In recent years, accumulating evidences showed that trehalose, serving as a signal molecule, plays very important role in plant growth, development and the acquisition of stress tolerance including heat tolerance (Paul et al., 2008; Fernandez et al., 2010). However, interaction of hydrogen sulfide (H_2_S) and trehalose in the acquisition of heat tolerance in plants still remains elusive.

Hydrogen sulfide, a colorless gas with a strong odor of rotten eggs, has long been considered as a phytotoxin (Zhang et al., [Bibr CR44][Bibr CR45]; Chen et al., 2011; Li et al., 2011; Li, 2013). However, in recent years, H_2_S has been identified as a third endogenous gaseous transmitter after nitric oxide (NO) and carbon monoxide (CO), playing multiple physiological roles in animal systems (Li et al., 2011; Wang, 2012). In plant systems, the positive effect of H_2_S is being emerged in growth, development, and the acquisition of stress tolerance (García-Mata and Lamattina, 2010; Hancock et al., 2011). Pretreatment with H_2_S donor NaHS can improve the resistance of plants to osmotic and oxidative stress in sweetpotato (Zhang et al., 2009a) and wheat (Zhang et al., 2011) by increasing the activity of the antioxidant enzymes. In addition, Shan et al. (2011) found that fumigation of spinach increased ascorbic acid and glutathione levels, and it was estimated that approximately 40% of the H_2_S was converted to glutathione in the leaves. Also, H_2_S donor, NaHS or p-(methoxyphenyl) morpholino-phosphin-odithioic acid (GYY 4137), can promote seed germination (Zhang et al., 2010b; Li et al., 2012a), root organogenesis (Zhang et al., 2009b), stomata opening (Lisjak et al., 2010), and improve the antioxidative response to heavy metal such as chromium stress (Zhang et al., 2010a). Our previous results also showed that NaHS treatment could improve the resistance of tobacco cells (Li et al., 2012b), maize (Li et al., [Bibr CR22][Bibr CR23]) and wheat seedlings (Wu et al., 2013) to high temperature and drought stress, and the acquisition of this stress tolerance involved in the second messenger such as Ca^2+^, nitric oxide (NO) and proline accumulation. But in maize seedlings, the mechanisms of H_2_S-induced heat tolerance are not fully clear.

Maize (*Zea mays* L.) not only is the third most important food grain crop after wheat and rice, but also a new model plant, high temperature is the principal cause of maize failure worldwide, global warming accentuates this problem (Leipner and Stamp, 2009; Strable and Scanlon, 2009). Many studies have showed high temperatures not only lead to direct injuries including protein denaturation and aggregation, increased fluidity of membrane lipids, but also to indirect heat injuries like cell dehydration, inactivation of enzymes, inhibition of protein synthesis and loss of membrane integrity, and eventually result in severe cellular injury and even cell death (Wahid et al., 2007; Hanumappa and Nguyen, 2010).

On the basis of the above-mention evidences, implying interaction between H_2_S and trehalose may be existed in the acquisition of stress tolerance including heat tolerance, but their interaction in the acquisition of heat tolerance in maize seedlings is unknown. In this study, using maize seedlings as materials, effect of NaHS treatment on heat tolerance and trehalose accumulation, as well as its possible metabolic pathways were investigated, the purpose was to expound interaction between H_2_S and trehalose in the acquisition of heat tolerance in maize seedlings.

## Methods

### Plant material, sodium hydrosulfide pretreatment and heat tolerance

Commercial variety of maize (*Zea mays* L., Huidan No. 4) was used in the present experiments. Like numerous previous researchers, NaHS was used as a donor for H_2_S, when NaHS is dissolved in water, HS^-^ is released and forms H_2_S with H^+^ (Wang, 2012). The seeds were sterilized in 0.1% HgCl_2_ for 10 min, and pre-soaked in distilled water for 12 h for imbibition. The soaked seeds were sowed on six layers of wetted filter papers in trays (24 cm × 16 cm, approximately 300 seeds per tray) with covers and germinated at 26°C in the dark for 2.5 d. After germination, the seedlings with unanimous growth were irrigated with 100 ml of 0 (control), 0.3, 0.6, 0.9 and 1.2 mM sodium hydrosulfide (NaHS) (the pH of the solution was adjusted to 6.0 with 1 M HCl), a hydrogen sulfide donor (Lisjak et al., 2013; Wang, 2012; García-Mata and Lamattina, 2013) for 6 h (pretreatment with NaHS had no significant effect on the growth of seedlings). Afterward, NaHS-treated seedlings in trays with covers were exposed to high temperature at 47°C in the dark (70% RH) for 15 h for heat stress. At the end of heat stress, electrolyte leakage of roots, MDA content in coleoptiles and survival percentage (%) were assayed, respectively, according to our previous methods (Li et al., 2012b). One cm root tips of seedlings were cut off and the percentage of electrolyte leakage from the tips was measured with a conductometer. In addition, the level of lipid peroxidation in coleoptiles of the seedlings was measured in terms of MDA content determined by thiobarbituric acid reaction. Electrolyte leakage and MDA content were expressed as %, and nmol g^-1^ FW, respectively.

In addition to electrolyte leakage and MDA content, after heat stress, seedlings were transferred to a climate chamber with 26°C and 100 μmol · m^-2^ · s^-1^ as well as 12 h photoperiod for a week for recovery and irrigated with 1/2 Hoagland solution daily. Survival percentage (%) was counted after recovery, and the seedlings that could regrow and become green during recovery were considered to have survived (Li et al., [Bibr CR22][Bibr CR23]).

### Measurement of H_2_S content

In order to understand effect of different concentrations of NaHS treatment on endogenous H_2_S content, H_2_S content in coleoptiles of the seedlings was determined according to the methods described by Christou et al. (2013) with modifications. Briefly, coleoptiles of maize seedlings (1 g) were ground into fine powder with a mortar and pestle under liquid nitrogen and then were homogenized in 1 ml of 100 mM potassium phosphate buffer (pH 7.0) containing 10 mM EDTA. The homogenate was centrifuged at 15,000 × *g* for 15 min at 4°C and 200 μl of the supernatant was used for the quantification of H_2_S, in an assay mixture containing also 3760 μl extraction buffer and 40 μl of 20 mM 5,5′-dithiobis (2-nitrobenzoic acid), in a total volume of 4 ml. The assay mixture was incubated at room temperature for 2 min and the absorbance was read at 412 nm. H_2_S was calculated based on a standard curve of known concentrations of NaHS and expressed as nmol g^-1^ fresh weight (FW).

### Measurement of trehalose-6-phosphate phosphatase and trehalase activities

Trehalose-6-phosphate phosphatase (TPP) and trehalase are key enzymes in trehalose acclimation in plants, the former is used for trehalose biosynthesis, while the latter controls its degradation (Paul et al., 2008). During the process of 0.6 mM NaHS treatment and heat stress, TPP and trehalase activities in coleoptiles and roots of maize seedlings were measured according to Lunn et al. (2006) and (El-Bashiti et al. 2005). Briefly, the samples of coleoptiles or roots (1 g) were ground with liquid nitrogen by using mortar and pestle. The powders were then suspended in ice-cold suspension solution containing 0.1 M citrate-Na^+^, pH 3.7, 1 mM PMSF, 2 mM EDTA and insoluble 1% (w/v) polyvinylpyrrolidone. The homogenate was centrifuged at 15,000 × *g* for 20 min at 4°C. The supernatant was used for TPP and trehalase assays .

TPP (TPP, EC 3.1.3.12) activity was determined by following the release of orthophosphate from Trehalose-6-phosphate (Tre6P). Reactions containing 1.25 mM Tre6P and 8 mM MgCl_2_ in 25 mM Hepes-K^+^, pH 7.0, in a total volume of 300 *μ* l were incubated at 30°C and stopped by adding 30 *μ* l of 2 M trichloroacetic acid. Orthophosphate was measured in samples using the ascorbic acid–ammonium molybdate reagent and TPP activity was expressed as *μ* mol g^-1^ FW min^-1^.

Trehalase (EC 3.2.1.28) activity was determined calorimetrically according to Garg and Chandel (2011) by measuring the glucose released. The reaction mixture contained 50 mM trehalose in 50 mM MES/KOH buffer (pH 6.3). After incubation at 30°C for 30 min, the reaction was stopped by heating at 100°C for 5 min. The glucose released was measured by the dinitrosulfosalicylic acid (DNSA) reagent. The trehalase activity was calculated using a standard curve of glucose and expressed as *μ* mol g^-1^ FW.

### Determination of trehalose content

To investigate effect of NaHS pretreatment on trehalose content under normal cultural conditions and heat stress, after being treated with solution of 0.6 mM NaHS alone or in combination with 0.6 mM sodium citrate (a inhibitor of TPP) for 0, 3, 6 and 9 h, the accumulation of trehalose in coleoptiles and roots was determined as per the methods of Kumar et al. (2012) as well as Jagdale and Grewal (2003) with modifications. The coleoptiles or roots (1 g) were homogenized in 5 ml of 80% (v/v) hot ethanol and centrifuged at 15,000 × *g* for 15 min, and the supernatants were transferred to glass tube and dried at 80°C. After drying, samples were redissolved with 5 ml distilled water as assay solution and used for assays of trehalose. Detailed protocols were described as follow: A 100 μl assay solution was mixed with 0.2 N H_2_SO_4_ and boiled at 100°C for 10 min to hydrolyze any sucrose or glucose-1-phosphate etc., and then chilled on ice. A 150 μl 0.6 N NaOH was added to above mixture and boiled for 10 min to destroy reducing sugars, and then chilled again. 2.0 ml of anthrone reagent (0.05 g anthrone per 100 ml of 72% H_2_SO_4_) was added and boiled for 10 min to develop a color and then chilled again. Then absorbance measurements were made at 630 nm with spectrophotometer. The trehalose concentration was calculated using a standard curve and expressed as nmol g^-1^ FW.

### Trehalose treatment and heat tolerance

To further study effect of the combination of trehalose and NaHS pretreatment on content of endogenous trehalose and heat tolrance of maize seedlings, 2.5-day-old seedlings were transferred to aqueous solution of 0 (control), 5, 10, 15, 20 and 25 mM trehalose alone in combination with 0.6 mM NaHS for 9 h, and the contents of endogenous trehalose and H_2_S in coleoptiles was measured as above-mentioned methods, respectively. In addition, seedlings treated with trehalose for 6 h were subjected to high temperature at 47°C for 15 h. Electrolyte leakage of roots, MDA content in coleoptiles and survival percentage were assayed according to above-mentioned methods, respectively.

### Statistical analysis

The experiment was set up according to a completely randomized design with five replications. The data were processed statistically using software package SPSS version 21.0 (SPSS, Chicago, USA) and the comparison of averages of each treatment was based on the analysis of variance (one-way ANOVA) according to Duncan’s multiple range test. Figures were drawn by SigmaPlot 12.0 (Systat Software Inc., London, UK), error bars represent standard error and each data in figure represents the mean ± SE of at least three independent experiments, asterisk and double asterisks on the bars indicate significant differences (*P* < 0.05) and very significant differences (*P* < 0.01).

## Results

### Effect of NaHS treatment on endogenous H_2_S content under normal culture conditions and heat tolerance in maize seedlings

**Figure 1:**
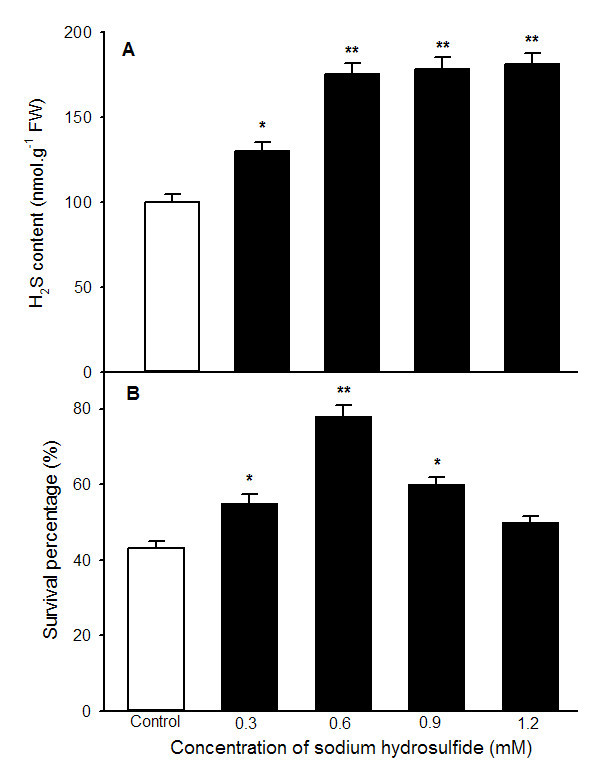
**Effects of NaHS pretreatment on the content of endogenous H**_**2**_**S (A) under normal culture conditions and survival percentage (B) under heat stress at 47°C.** Seedlings of maize were irrigated with 100 ml of 0 (control), 0.3, 0.6, 0.9 and 1.2 mM NaHS for 6 h, respectively, and then determined the content of endogenous H_2_S in coleoptiles of maize seeds. Afterwards, treated seedlings were subjected to heat stress at 47°C for 15 h and survival percentage of seedlings was counted. Error bars represent standard error and each data in figure represents the mean ± SE of three experiments, and asterisk and double asterisks indicate significant difference (*P* < 0.05) and very significant difference (*P* < 0.01) from the control without NaHS treatment, respectively.

2.5-day-old maize seedlings was subjected to heat stress at 47°C for 15 h after being treated with 0 (control), 0.3, 0.6, 0.9 and 1.2 mM NaHS for 6 h. As shown in Figure 1, under normal culture conditions at 26°C, application of NaHS with different concentrations, compared with the control without NaHS treatment, improved endogenous H_2_S content in coleoptiles of maize seedlings, especially in pretreatment with 0.6 mM and above reached very significant difference (*P* < 0.01, Figure 1A), which in turn increased survival percentage of maize seedlings under heat stress at 47°C, and treatment with 0.6 mM reached very significant difference (*P* < 0.01, Figure 1B), similar to the content of endogenous H_2_S (Figure 1A), but higher concentration of NaHS (≥0.9 mM) reduced survival percentage (Figure 1B). Higher concentration of NaHS was bad for the acquisition of heat tolerance in seedlings of maize, coinciding with one of the characteristics of other signal molecules such as Ca^2+^, NO and H_2_O_2_.

**Figure 2:**
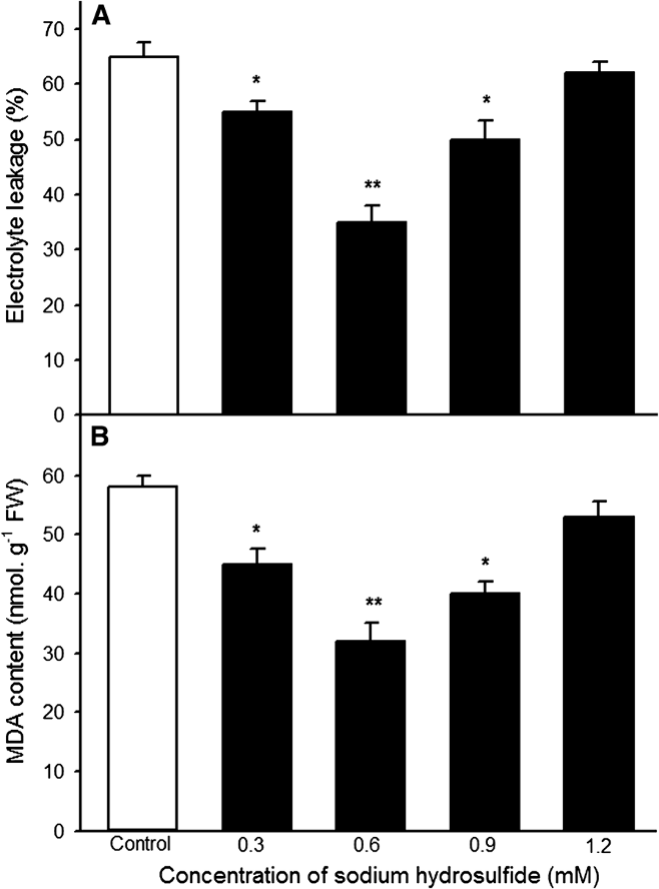
**Effects of NaHS pretreatment on electrolyte leakage (A) and malonaldehyde (MDA) content (B) under heat stress at 47°C.** Seedlings of maize were irrigated with 100 ml of 0 (control), 0.3, 0.6, 0.9 and 1.2 mM NaHS for 6 h, respectively, and then subjected to heat stress at 47°C for 15 h, the electrolyte leakage in roots and MDA content in coleoptiles was determined. Error bars represent standard error and each data in figure represents the mean ± SE of three experiments, and asterisk and double asterisks indicate significant difference (*P* < 0.05) and very significant difference (*P* < 0.01) from the control without NaHS treatment, respectively.

Heat stress damages membrane systems and leads to the leakage of electrolytes from plant cells, whereas MDA content is often used as a measure of lipid peroxidation as a result of reactive oxgen species (Wahid et al., 2007). In present experiments, to understand effect of different concentrations of NaHS treatment on electrolyte leakage and MDA content, which were determined and found that NaHS treatment alleviated increase in electrolyte leakage and MDA content, especially 0.6 mM NaHS treatment showed more significant difference (*P* < 0.01, Figure 2), but mitigative effect of higher concentrations of NaHS on heat stress was not obvious, analogous to change in survival percentage (Figure 1B). Therefore, the concentration of 0.6 mM NaHS was used in further experiments. These results illustrated that NaHS-induced heat tolerance of maize seedlings in a concentration-dependent manner may be achieved by accumulating endogenous H_2_S.

### Effect of NaHS on TPP and trehalose activities as well as endogenous trehalose content in maize seedlings

**Figure 3:**
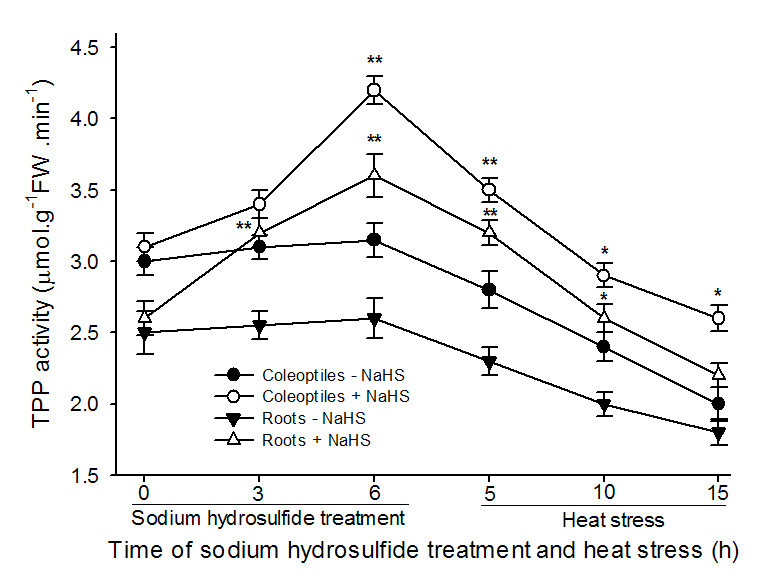
**Effects of NaHS pretreatment on the activity of trehalose 6-phosphate phosphatase (TPP) in coleoptiles and roots of maize seedlings under normal culture conditions and heat stress.** Seedlings of maize were irrigated with 100 ml of 0.6 mM NaHS for 6 h, and then transferred to heat stress, during the treatment and heat stress, TPP activity in both coleoptiles and roots was determined. Error bars represent standard error and each data in figure represents the mean ± SE of three experiments, and asterisk and double asterisks indicate significant difference (*P* < 0.05) and very significant difference (*P* < 0.01) from the control without NaHS treatment, respectively.

**Figure 4:**
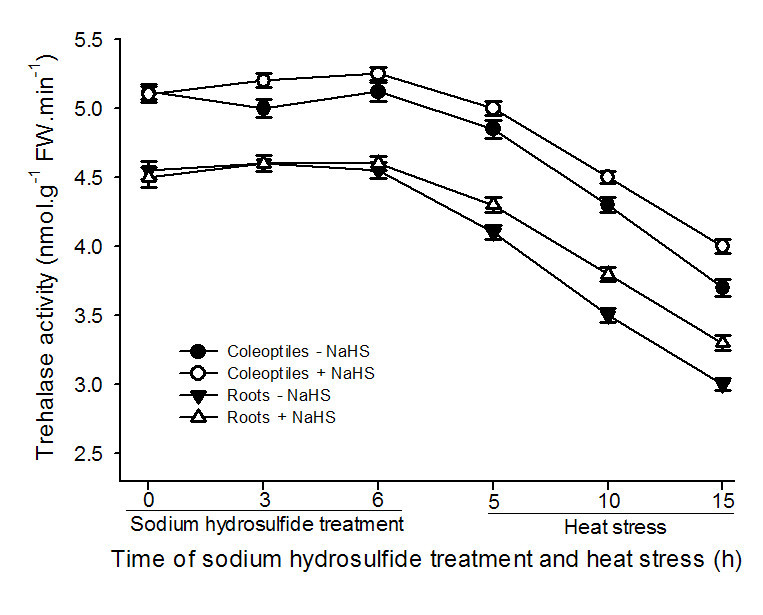
**Effects of NaHS pretreatment on the activity of trehalase in coleoptiles and roots of maize seedlings under normal culture conditions and heat stress.** Seedlings of maize were irrigated with 100 ml of 0.6 mM NaHS for 6 h, and then transferred to heat stress, during the treatment and heat stress, trehalase activity in both coleoptiles and roots was determined. Error bars represent standard error and each data in figure represents the mean ± SE of three experiments.

**Figure 5:**
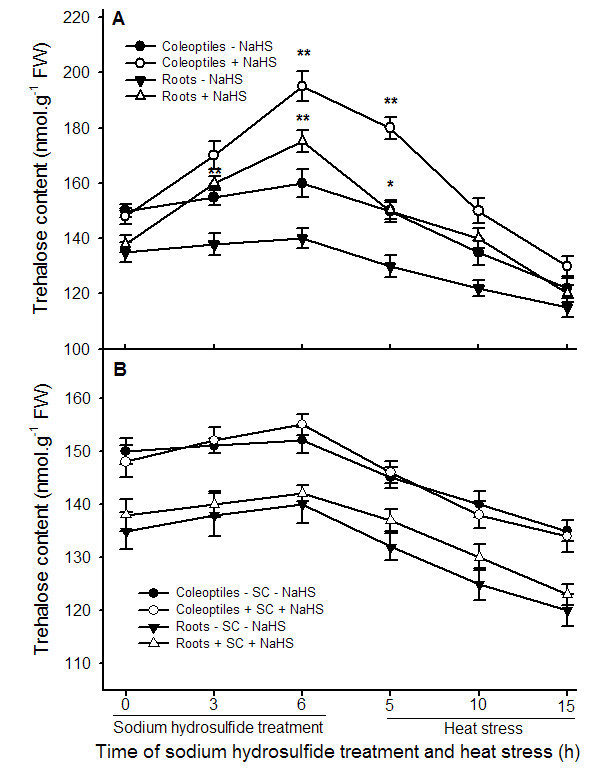
**Effects of NaHS (A) and TPP inhibitor sodium citrate (SC, B) pretreatment on the content of endogenous trehalose in coleoptiles and roots of maize seedlings under normal culture conditions and heat stress.** Seedlings of maize were irrigated with 100 ml of 0.6 mM NaHS alone or in combination with 0.6 mM sodium citrate for 6 h, and then transferred to heat stress, during the treatment and heat stress, endogenous trehalose content in coleoptiles and roots of maize seeds was determined. Error bars represent standard error and each data in figure represents the mean ± SE of three experiments, and asterisk and double asterisks indicate significant difference (*P* < 0.05) and very significant difference (*P* < 0.01) from the control without NaHS treatment, respectively.

To explore effect of NaHS on TPP and trehalase activity as well as the content of endogenous trehalose, after maize seedlings were treated with 0.6 mM NaHS, the activities of TPP and trehalase as well as the endogenous trehalose content were measured during the course of treatment and heat stress. The data exhibited that pretreatment with NaHS increased the activity of TPP in coleoptiles and roots of maize seedlings under normal cultural conditions, and this improvement increased with the increase of treatment time, 3 and 6 h of treatment reached very significant difference (*P* < 0.01, Figure 3) in roots and coleoptiles, respectively, while the activity of trehalase was not obvious from beginning to end (Figure 4). In addition, application of NaHS improved the content of endogenous trehalose in roots and coleoptiles of the seedlings under normal cultural conditions, reaching very significant difference (*P* < 0.01, Figure 5A) at 3 and 6 h of treatment, respectively, similar to the change in activity of TPP (Figure 3). The speed of trehalose accumulation in roots was more rapid than that of coleoptiles (Figure 5A), reaching very significant difference at 3 h of treatment in roots, but the amount of trehalose lowered than that of coleoptiles, analogous to the increase in the activity of TPP (Figure 3), while the accumulation of trehalose was eliminated by additon of sodium citrate, an inhibitor of TPP (Figure 5B). By contrast, during the process of heat stress, there was a trend toward decrease in TPP and trehalase activity as well as trehalose content in control and treated maize seedlings, but seedlings treated 0.6 mM NaHS maintained higher enzymes activities and trehalose content than those of control from beginning to end (Figures 3, 4 and 5). These results showed that pretreatment with NaHS could improve the content of endogenous trehalose under normal cultural conditions and alleviated decrease in its content under heat stress in maize seedlings, and this improvement and alleviation may be achieved by activating TPP activity.

### Effect of exogenous trehalose pretreatment on endogenous trehalose and H_2_S contents as well as heat tolerance in maize seedlings

**Figure 6:**
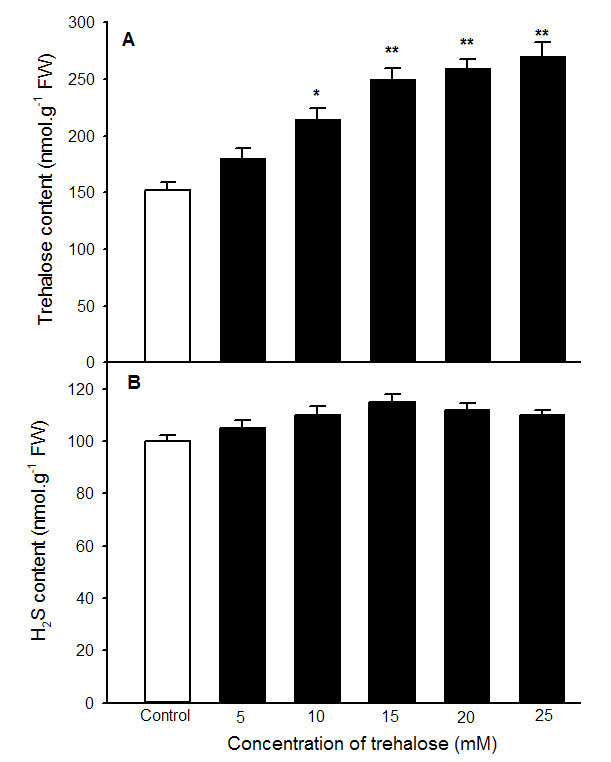
**Effects of exogenous trehalose pretreatment on the content of endogenous trehalose (A) and H**_**2**_**S (B) under normal culture conditions.** Seedlings of maize were irrigated with 100 ml of 0 (control), 5, 10, 15, 20 and 25 mM trehalose for 6 h, respectively, and then determined the contents of endogenous trehalose and H_2_S in coleoptiles of maize seeds. Error bars represent standard error and each data in figure represents the mean ± SE of three experiments, and asterisk and double asterisks indicate significant difference (*P* < 0.05) and very significant difference (*P* < 0.01) from the control without trehalose treatment, respectively.

**Figure 7:**
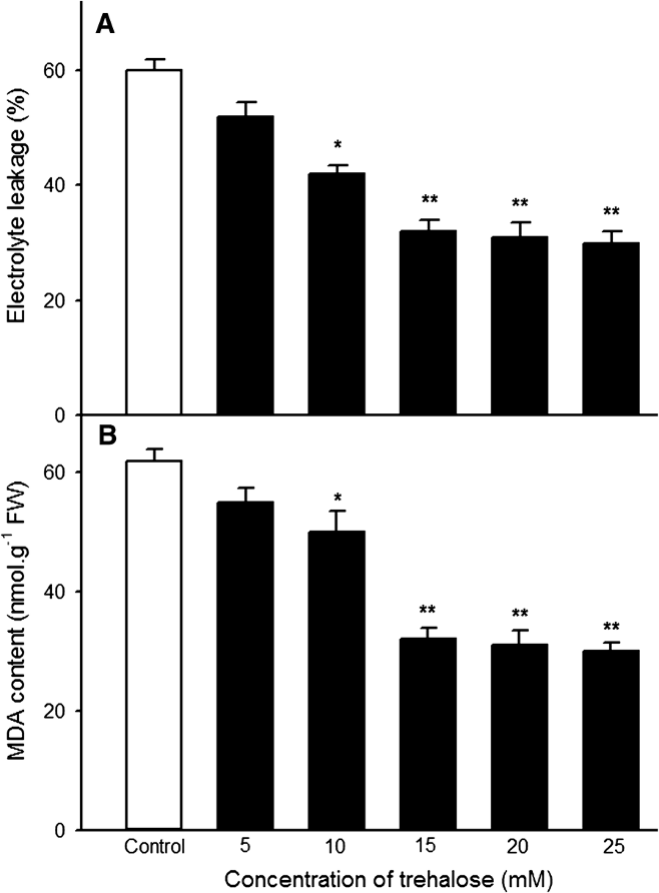
**Effects of exogenous trehalose pretreatment on electrolyte leakage (A) and the content of MDA (B) of maize seedlings under heat stress at 47°C.** were irrigated with 100 ml of 0 (control), 5, 10, 15, 20 and 25 mM trehalose for 6 h, respectively, and then were subjected to heat stress at 47°C for 15 h, electrolyte leakage in roots and MDA content in coleoptiles of maize seedlings were calculated. Error bars represent standard error and each data in figure represents the mean ± SE of three experiments, and asterisk and double asterisks indicate significant difference (*P* < 0.05) and very significant difference (*P* < 0.01) from the control without trehalose treatment, respectively.

**Figure 8:**
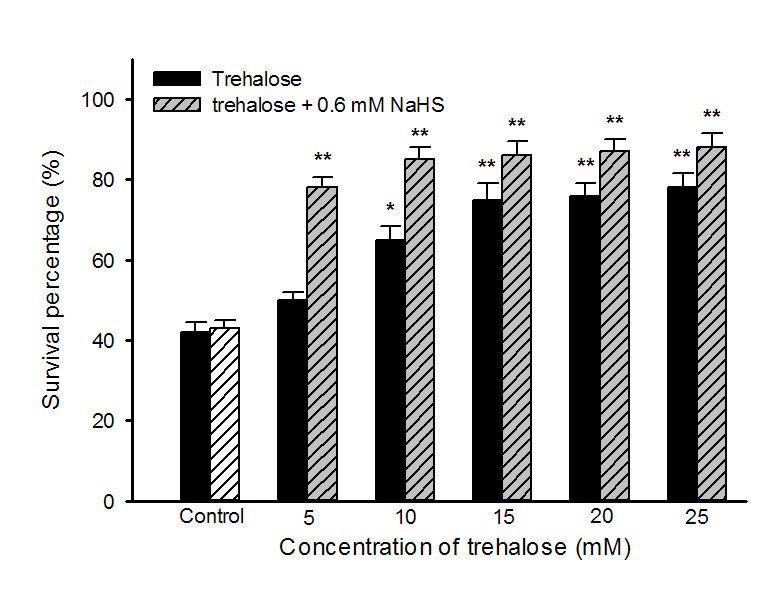
**Effects of exogenous trehalose pretreatment alone or in combination with 0.6 mM NaHS on survival percentage of maize seedlings under heat stress at 47°C.** Seedlings of maize were irrigated with 100 ml of 0 (control), 5, 10, 15, 20 and 25 mM trehalose alone or in combination with 0.6 mM NaHS for 6 h, respectively, and then were subjected to heat stress at 47°C for 15 h, the survival percentage of maize seedlings were calculated. Error bars represent standard error and each data in figure represents the mean ± SE of three experiments, and asterisk and double asterisks indicate significant difference (*P* < 0.05) and very significant difference (*P* < 0.01) from the control without trehalose treatment, respectively.

Pretreatment with NaHS could increase accumulation of endogenous trehalose (Figure 5A), which in turn improve heat tolerance of maize seedlings (Figures 1 and 2). To investigate effect of exogenous application of trehalose on the content of endogenous trehalose and H_2_S as well as heat tolerance, endogenous trehalose and H_2_S contents, electrolyte leakage, MDA content and survival percentage were determined after maize seedlings were treated with different concentration of trehalose alone or in combination with 0.6 mM NaHS. The results indicated that exogenously applied trehalose could increase content of endogenous trehalose in coleoptiles under normal conditions (Figure 6A), and alleviated increase in electrolyte leakage in roots and MDA accumulation in coleoptiles of seedlings under heat stress (Figure 7), which in turn improved survival percentage of maize seedlings (Figure 8). The content of endogenous trehalose and survival percentage improved along with the increase in concentration of exogenous trehalose (Figures 6A, and 8), while electrolyte leakage and MDA content decreased with increasing concentration of trehalose (Figure 7). The change in these parameters reached very significant difference (*P* < 0.01, Figures 6, 7 and 8), respectively, when the concentration of exogensous trehalose was ≥ 15 mM. In addition, exogenous trehalose treatment had not significant effect on endogenous H_2_S level (Figure 6B), but the heat tolerance induced by trehalose was enhanced by supplement of NaHS (Figure 8). These results displayed that pretreatment with exogenous trehalose could improve heat tolerance of maize seedlings and the acquisition of this heat tolerance was the outcome of accumulation of endogenous trehalose.

## Discussion

Accumulating evidences showed that H_2_S is an important signaling molecule involved in modulation of many physiological processes in plants (Mancardi et al., 2009; Zhang et al., [Bibr CR44][Bibr CR45]; Chen et al., 2011; Wang, 2012; Li, 2013; Li et al., 2013b). In the work of (García-Mata and Lamattina 2010) displayed that H_2_S participated in the abscisic acid (ABA)-dependent signalling pathway in guard cells of *Vicia faba* (L), which in turn induced stomatal closure, followed by protected plants against drought stress. Zhang et al. (2008; [Bibr CR44][Bibr CR45]; 2011) found that NaHS treatment could significantly reduce MDA and H_2_O_2_ accumulation in wheat seeds by enhancing the activities of antioxidant enzymes catalase and ascorbate peroxidase, which in turn improved germination percentage of wheat seeds under normal conditions or multiple abiotic stress such as PEG, Cu^2+^, Cr, and Al^3+^. Interestingly, in *Caenorhabditis elegans*, H_2_S treatment can increase thermotolerance and lifespan (Miller and Roth, 2007). Our previous results also found that NaHS pretreatment could improve heat tolerance in tobacco suspension cultured cells and wheat seedlings, and the acquisition of this heat tolerance requires the entry of extracellular Ca^2+^ into cells across the plasma membrane and the mediation of intracellular calmodulin (Li et al., 2012b; Wu et al., 2013). In addition, NaHS pretreatment markedly improved the activity of Δ^1^-pyrroline-5-carboxylate synthetase (P5CS) and lowered proline dehydrogenase (ProDH) activity in maize seedlings, which in turn induced the accumulation of endogenous proline, followed by improved germination percentage of seeds and survival percentage of seedlings of maize under heat stress (Li et al., 2013a), indicating that the acquisition of heat tolerance associated closely with multiple functions of proline such as maintaining integration of biomembrane and compartmentalization of organs, osmotic adjustment, redox buffering, and ROS-scavenging, inconsistent with the results of Lv et al. (2011) in Arabidopsis seedlings under heat stress. A further study observed that the acquisition of heat tolerance induced by H_2_S may be required crosstalk among second messengers such Ca^2+^, NO, H_2_O_2_ (Li et al., 2013b). In present study, NaHS pretreatments could increase endogenous H_2_S content in maize seedlings under normal conditions, which in turn improved heat tolerance of seedlings of maize (Figures 1 and 2). All of the above-mentioned studies illustrated that pretreatment with H_2_S could improve multiple stress tolerance such as heat tolerance, and the acquisition of stress tolerance may be involved in enhancement of antioxidant enzymes and accumulation of osmolyte proline by interactions among second messengers such Ca^2+^, NO, H_2_O_2_, but whether the acquisition of heat tolerance of maize seedlings involved in the accumulation of trehalose is still elusive.

As mentioned above, there are two pathways of trehalose biosynthesis in higher plants, that is, the trehalose-6-phosphate synthase/phosphatase (OtsA-OtsB) and the trehalase pathway, the former serves as trehalose biosynthesis, while the latter degrades it (Paul et al., 2008; Fernandez et al., 2010). Due to multiple functions of trehalose, similar to proline, such as stabilizing enzymes, proteins and lipid membranes; preventing protein aggregation; protecting biological structures; and scavenging free radical, numerous studies showed that trehalose involves in the acquisition of multiple stress tolerance (Goddijn and van Dun, 1999; Iordachescu and Imai, 2008; Paul et al., 2008; Fernandez et al., 2010). In rice seedlings, the transgenic plants with trehalose biosynthetic genes (*otsA* and *otsB*) accumulate trehalose at levels 3 ~ 10 times that of the nontransgenic controls, and this accumulation correlates with higher soluble carbohydrate levels, which in turn increased the resistance of plants to salt, drought and low-temperature stress (Garg et al., 2002). Similarly, under drought, salt and oxidative stress, TPS1 tomato plants increased trehalose biosynthesis, followed by improved tolerance than wild type (Cortina and Culiáñez-Macià, 2005). In addition, transgenic tobacco and sugarcane with trehalose synthase (*Tsase)* accumulated more trehalose than the control, which in turn improved the contents of water, chlorophyll a and b as well as the activity of superoxide dismutase and peroxidase, finally resulted in increased tolerance to drought and salt (Zhang et al., 2005,2006). Luo et al. (2010) found that heat stress induced increase in endogenous trehalose, which in turn improved the resistance of winter wheat to heat stress by lowering the electrolyte leakage, content of MDA, generation of ROS, and lipoxygenase activity. In present study, pretreatment with NaHS increased the TPP activity in coleoptiles and roots of maize seedlings (Figure 3), which in turn improved the endogenous trehalose content in coleoptiles of the seedlings (Figure 5A), followed by elevated heat tolerance of maize seedlings (Figures 1 and 2), but exogenous application of NaHS had not significant effect on trehalase activity (Figure 4), indicating trehalose accumulation induced by NaHS was achieved by activating TPP. To further identify the acquisition of stress tolerance assuredly involved in trehalose, Ma et al. (2013) treated wheat callus with 50 mM trehalose and found that pretreatment with trehalose enhanced levels of endogenous trehalose, alleviated the accumulation of MDA and the generation of ROS induced by water deficit, which in turn resulted in elevating cell viability and biomass. Additionally, exogenous application of 10 μM trehalose promoted the growth in heat-stressed plants and trehalose functions downstream of ABA (Kumar et al., 2012). In present work, exogenously applied trehalose could elevate endogenous trehalose content in coleoptiles under normal conditions (Figure 6A), and alleviated increase in electrolyte leakage in roots and MDA accumulation in coleoptiles of seedlings under heat stress (Figure 7), which in turn improved survival percentage of maize seedlings (Figure 8). In addition, the heat tolerance induced by trehalose was enhanced by exogenous supplement of NaHS, whereas exogenous trehalose treatment had not significant effect on the accumulation of endogenous H_2_S in maize seedlings (Figures 6B and 8). These results display that trehalose may be exerted role in downstream of H_2_S in the acquisition of stress tolerance including heat tolerance (Goddijn and van Dun, 1999; Benaroudj et al., 2001; Iordachescu and Imai, 2008; Luo et al., 2008; Paul et al., 2008; Fernandez et al., 2010). In present experiments, osmotic adjustment role of trehalose may be unimportant due to its lower accumulation (approximately 200 ~ 250 nmol g^-1^ FW). Therefore, the acquisition of heat tolerance induced by NaHS in maize seedlings may be an outcome of combined action of antioxidant defense system (Zhang et al., 2008; 2010a, b; 2011) and small molecule chaperones such as proline (Li et al. 2013a) and trehalose (Figures 1, 2, 3, 4, 5, 6 and 7).

## Conclusion

In summary, these data suggest that sodium hydrosulfide pretreatment could improve heat tolerance of maize seedlings in a concentration-dependent manner and this improvement may be involved in trehalose accumulation by activating TPP activity.

## Authors’ original submitted files for images

Below are the links to the authors’ original submitted files for images. Authors’ original file for figure 1 Authors’ original file for figure 2 Authors’ original file for figure 3 Authors’ original file for figure 4 Authors’ original file for figure 5 Authors’ original file for figure 6 Authors’ original file for figure 7 Authors’ original file for figure 8

## Abbreviations

ABA: Abscisic acid
G6P: Glucose 6-phosphate
MDA: Malonaldehyde
P5CS: Δ^1^-Pyrroline-5-carboxylate synthetase
ProDH: Proline dehydrogenase
ROS: Reactive oxygen species
T6P: Trehalose 6-phosphate
TPP: Trehalose-6-phosphate phosphatase
TPS: Trehalose 6-phosphate synthase
UDP: Uridine diphosphate
UDPG: Uridine diphosphate glucose.
