# Supplementary figures and images for: Involvement of trehalose in hydrogen sulfide donor sodium hydrosulfide-induced the acquisition of heat tolerance in maize (Zea mays L.) seedlings
Source: Bot Stud. 2014 Feb 3;55:20. doi: 10.1186/1999-3110-55-20 (PMC5432828; doi:10.1186/1999-3110-55-20)

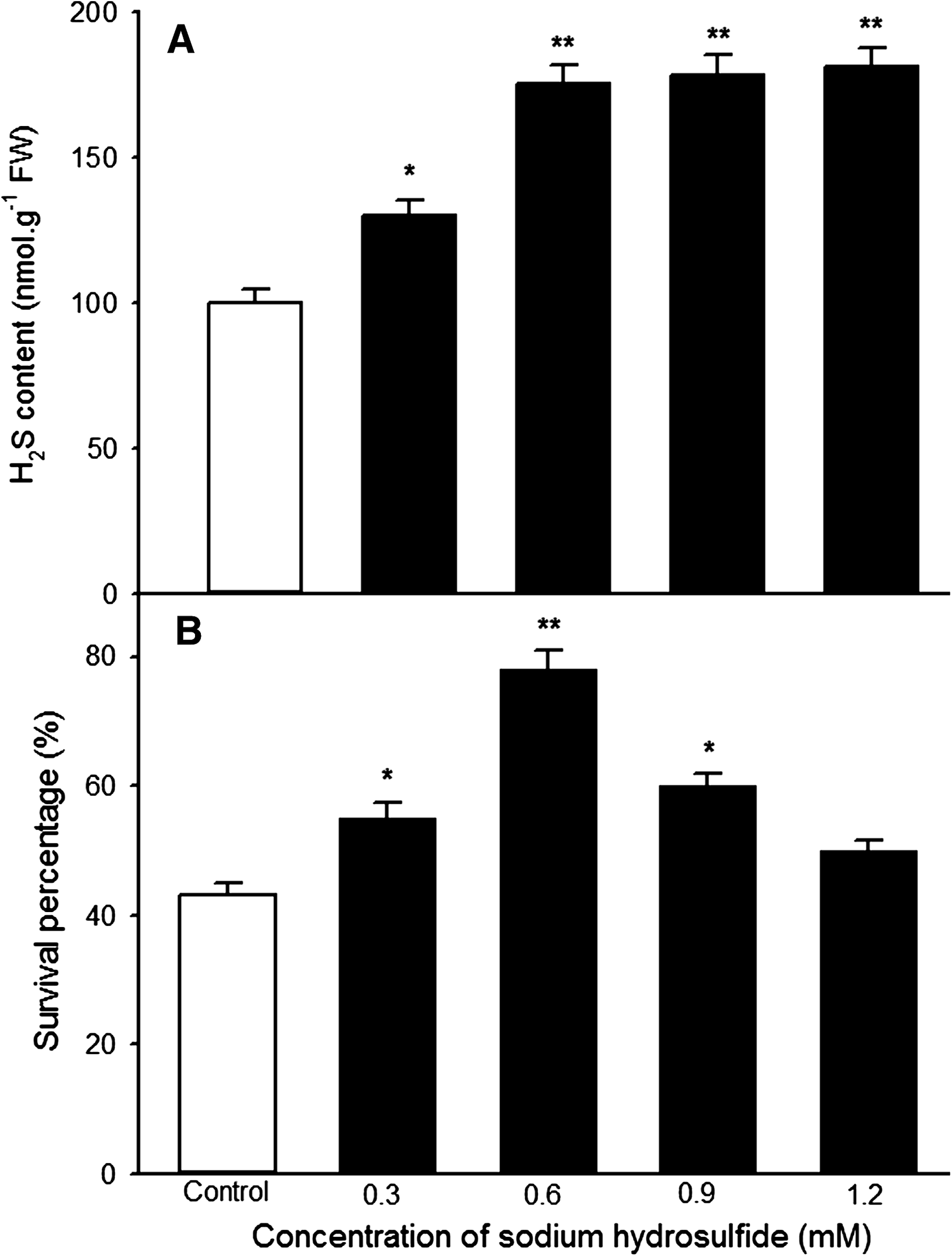

Supplement: Supplementary file 1 — Authors’ original file for figure 1 [file 40529_2013_65_MOESM1_ESM.tif]

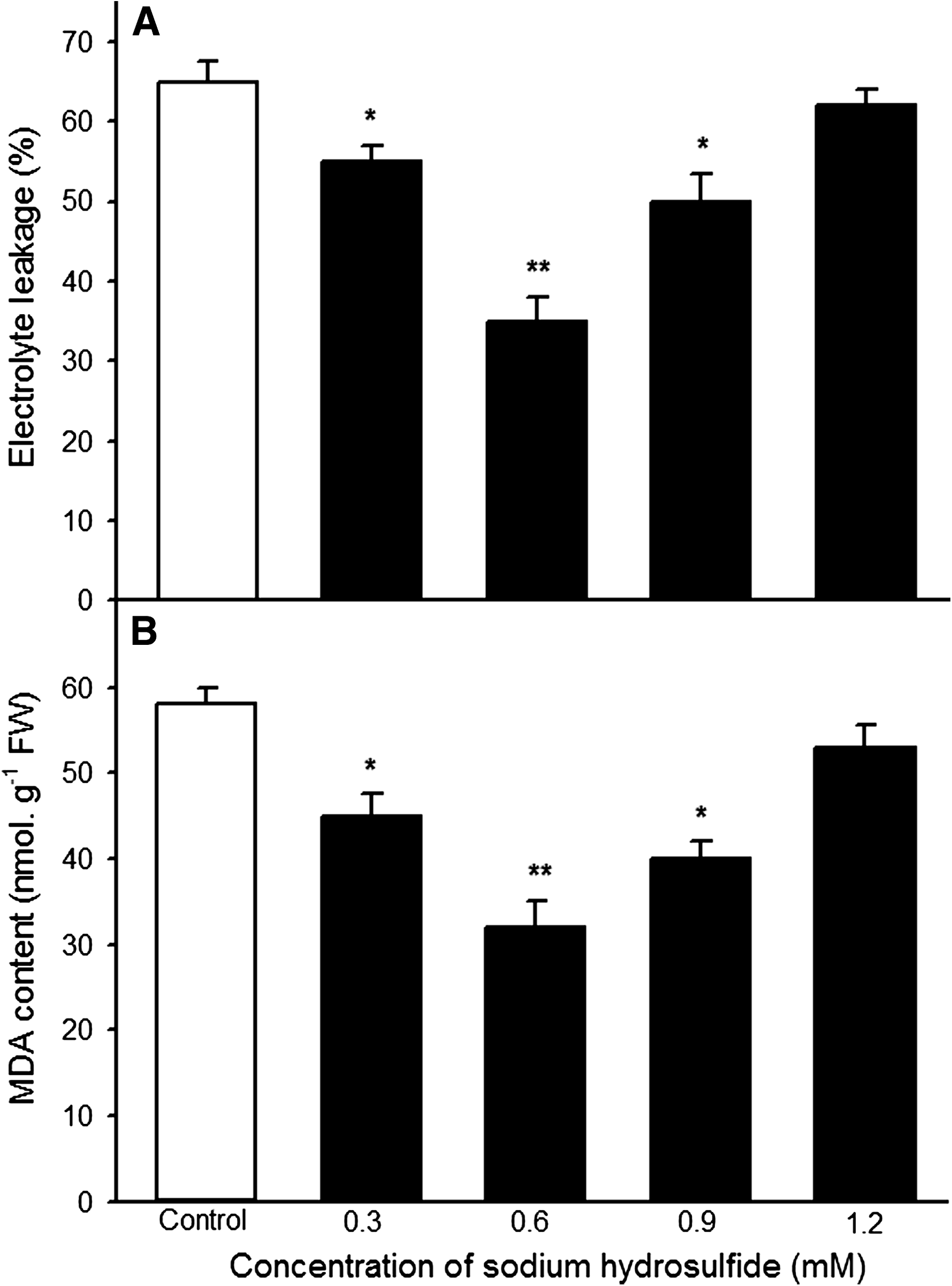

Supplement: Supplementary file 2 — Authors’ original file for figure 2 [file 40529_2013_65_MOESM2_ESM.tif]

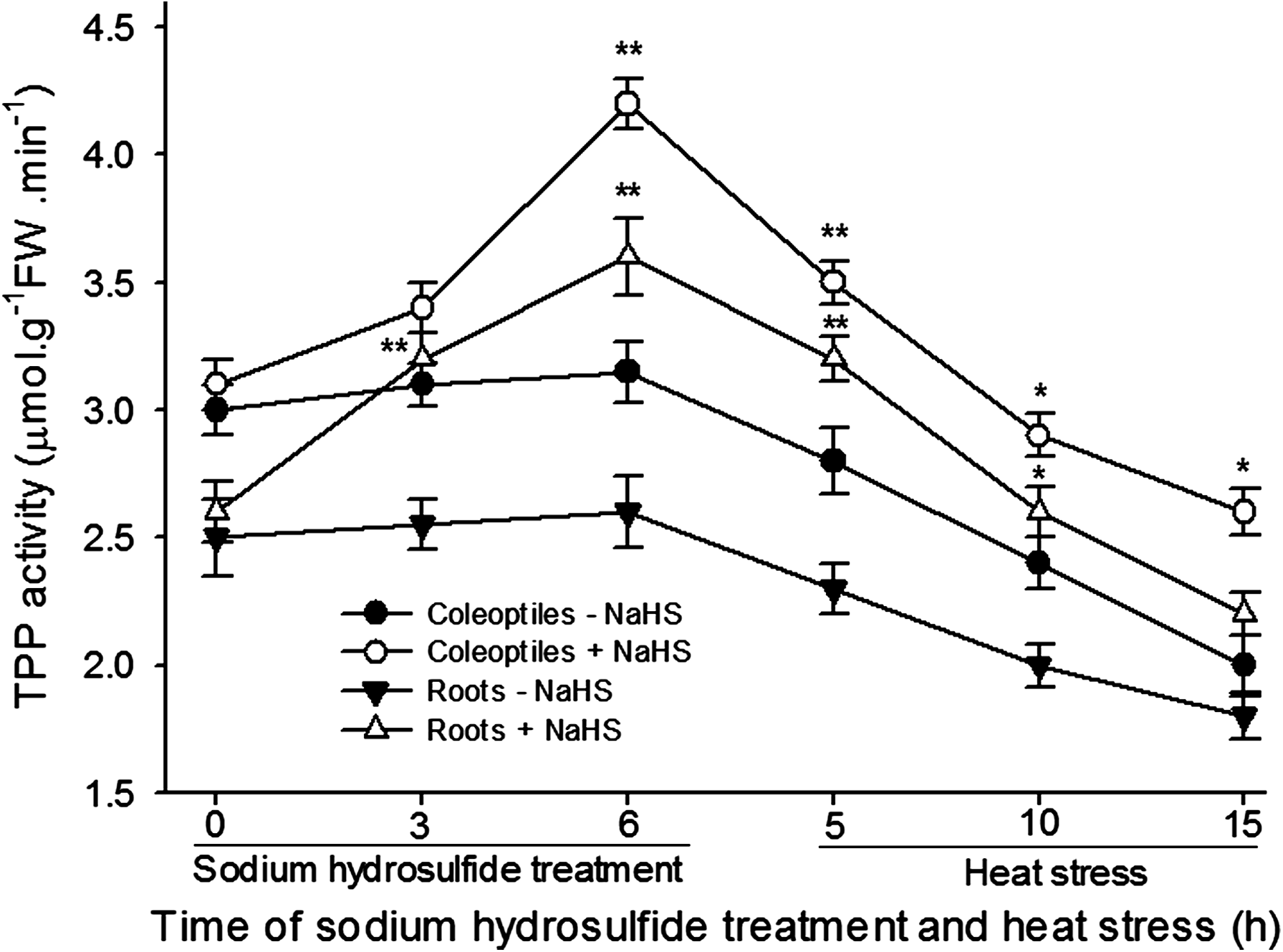

Supplement: Supplementary file 3 — Authors’ original file for figure 3 [file 40529_2013_65_MOESM3_ESM.tif]

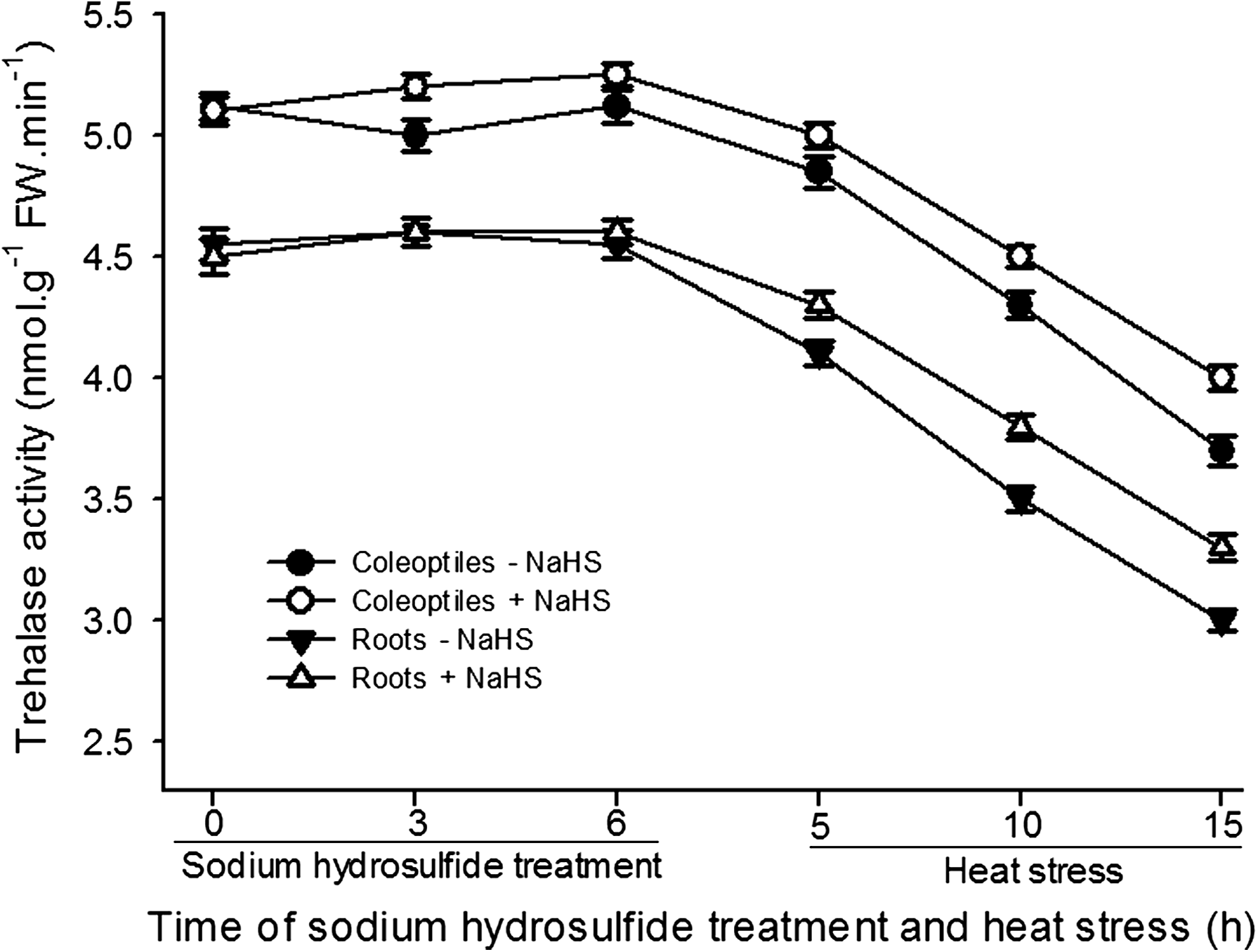

Supplement: Supplementary file 4 — Authors’ original file for figure 4 [file 40529_2013_65_MOESM4_ESM.tif]

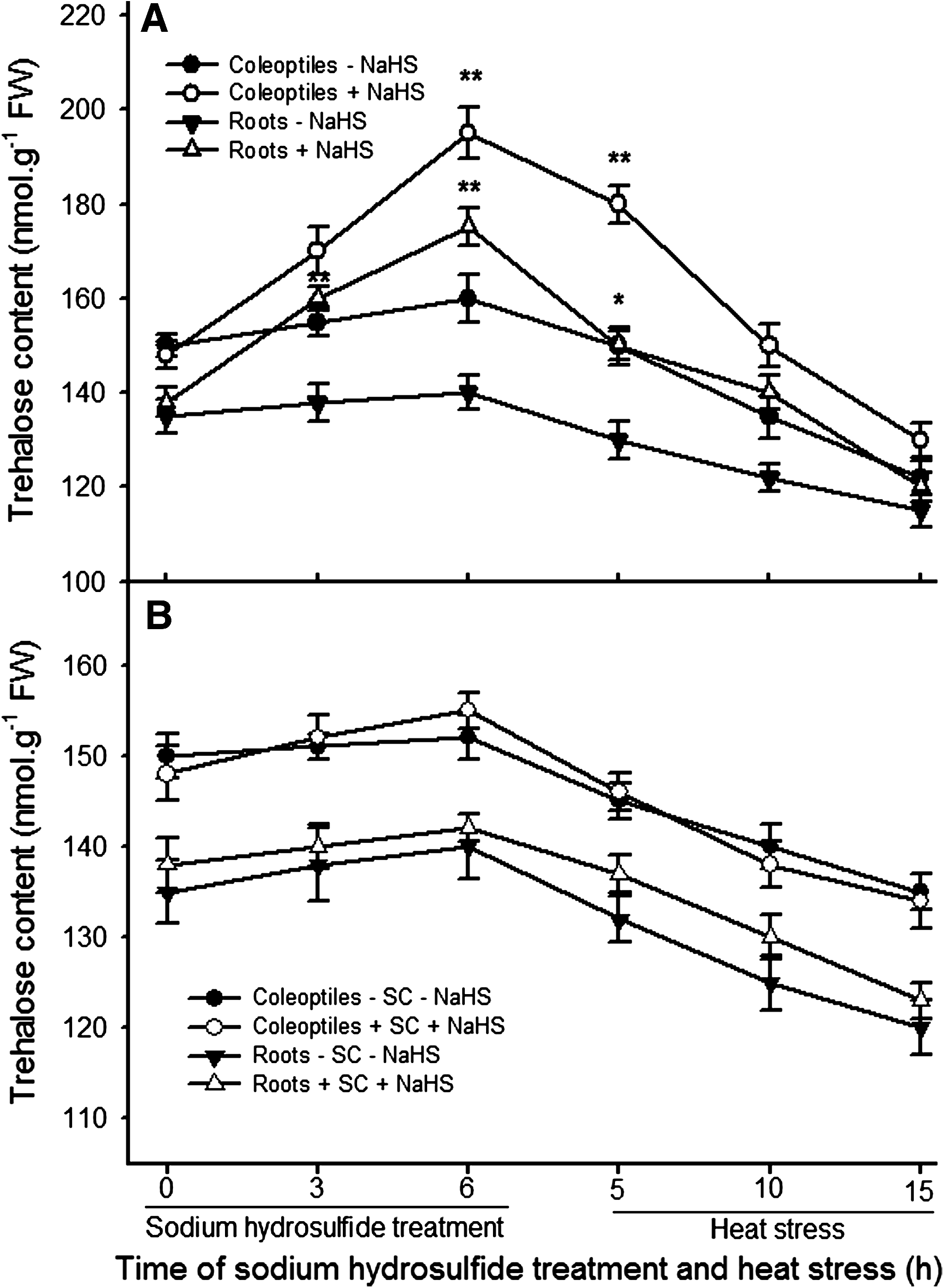

Supplement: Supplementary file 5 — Authors’ original file for figure 5 [file 40529_2013_65_MOESM5_ESM.tif]

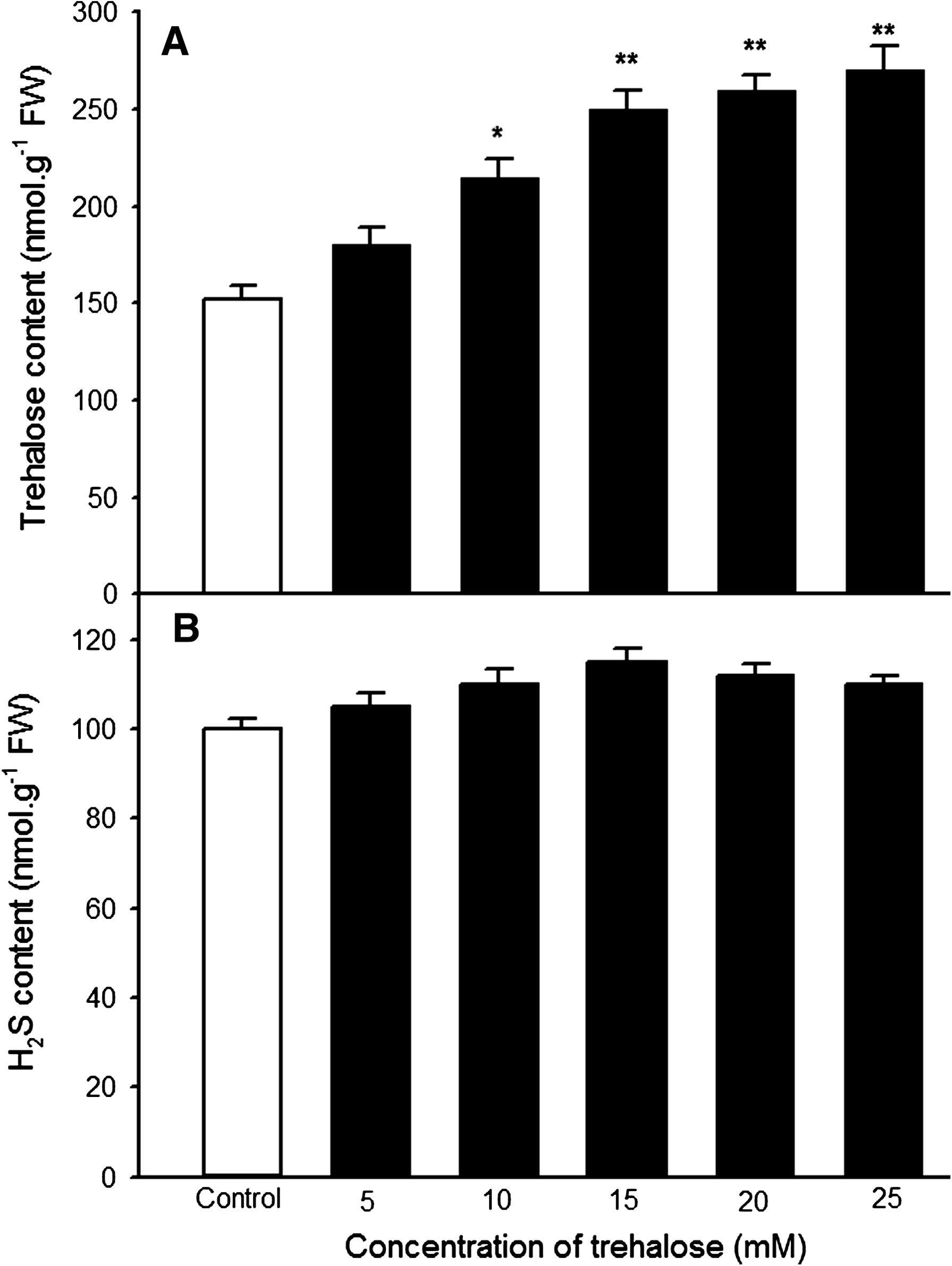

Supplement: Supplementary file 6 — Authors’ original file for figure 6 [file 40529_2013_65_MOESM6_ESM.tif]

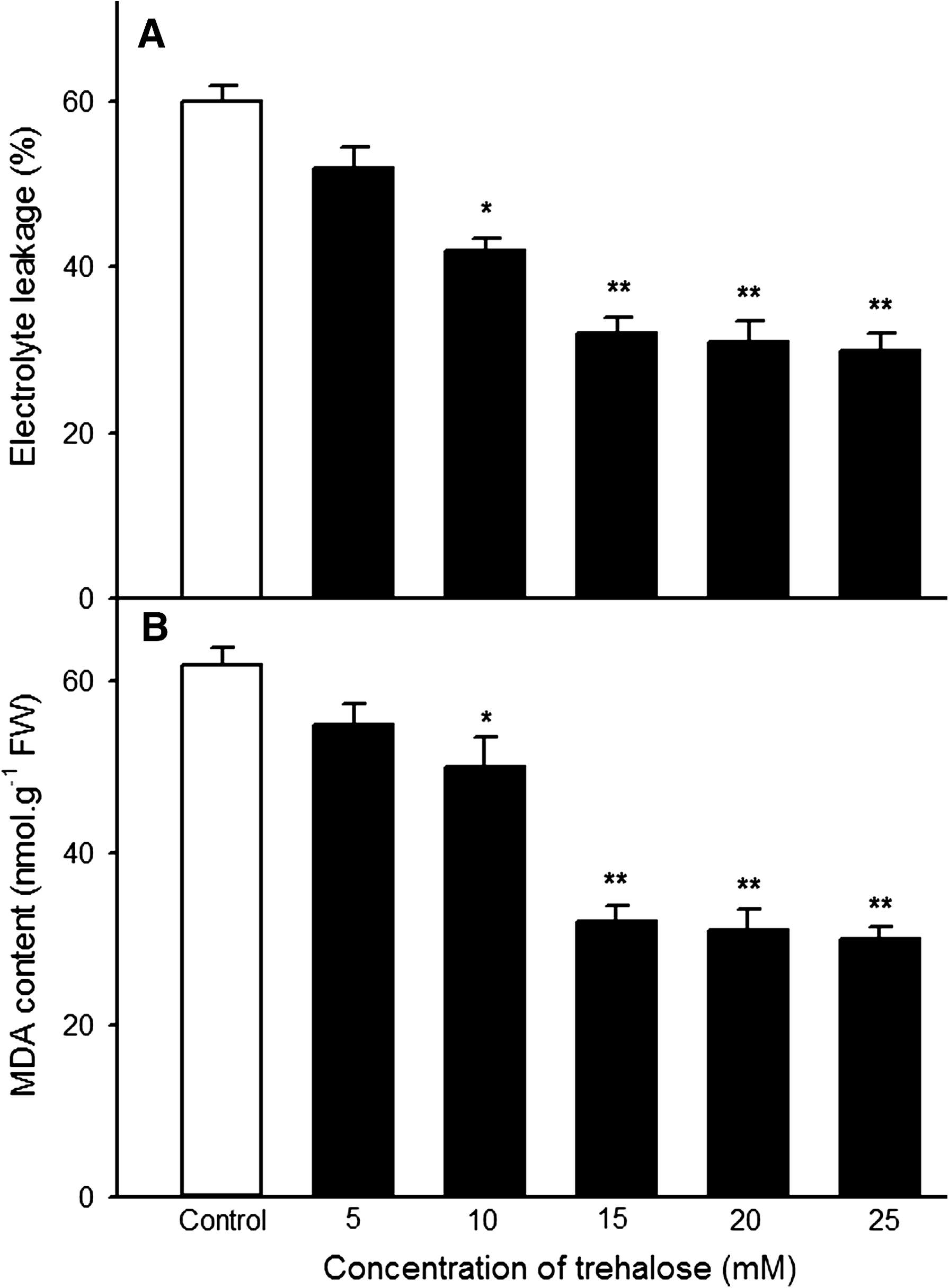

Supplement: Supplementary file 7 — Authors’ original file for figure 7 [file 40529_2013_65_MOESM7_ESM.tif]

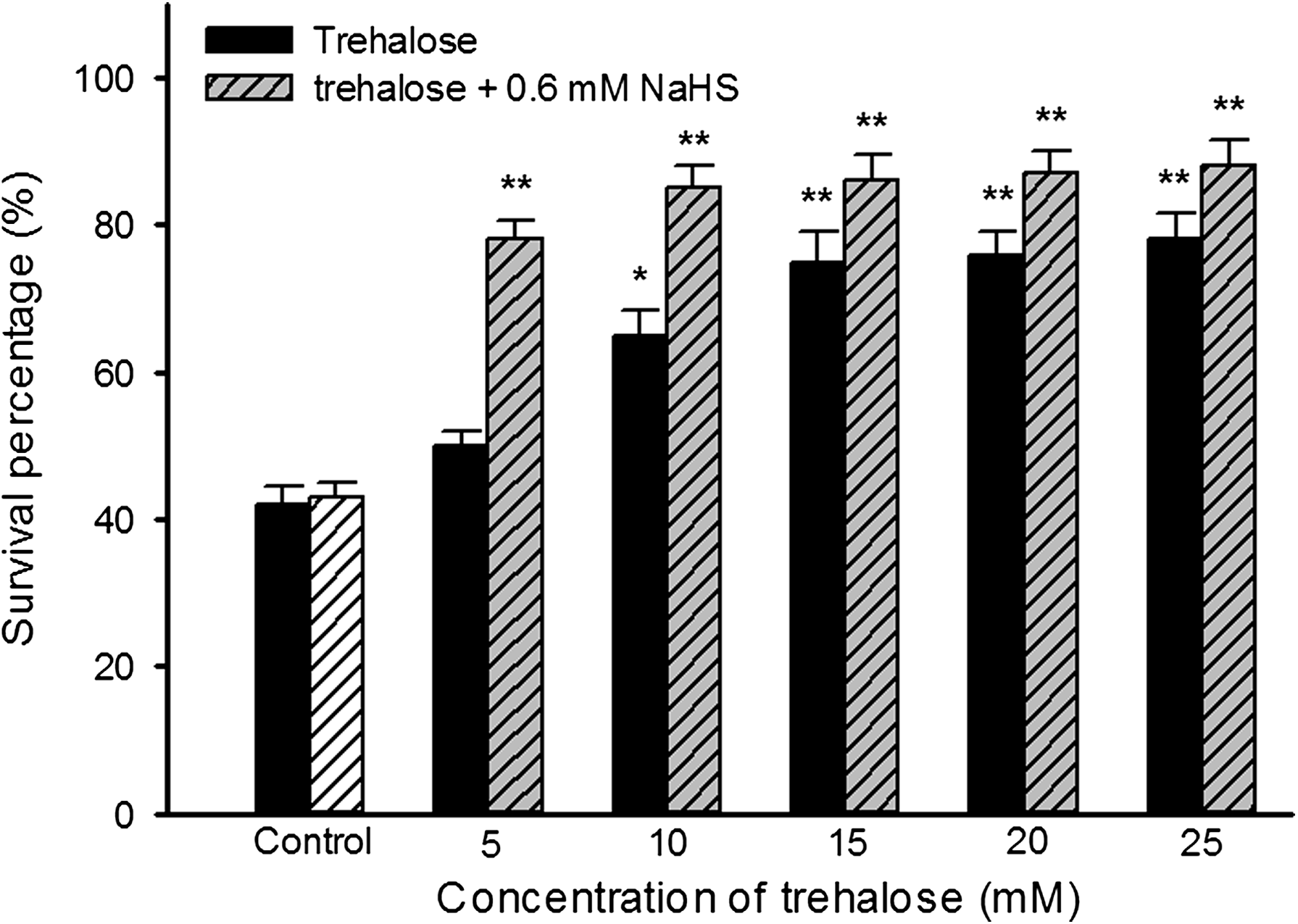

Supplement: Supplementary file 8 — Authors’ original file for figure 8 [file 40529_2013_65_MOESM8_ESM.tif]
